# A randomised controlled trial of compression therapies for the treatment of venous leg ulcers (VenUS 6): study protocol for a pragmatic, multicentre, parallel-group, three-arm randomised controlled trial

**DOI:** 10.1186/s13063-023-07349-2

**Published:** 2023-05-26

**Authors:** C. E. Arundel, C. Welch, P. Saramago, U. Adderley, R. Atkinson, I. Chetter, N. Cullum, T. Davill, J. Griffiths, C. Hewitt, C. Hirst, M. Kletter, J. Mullings, G. Roberts, B. Smart, M. Soares, P. Stather, L. Strachan, N. Stubbs, D. J. Torgerson, J. Watson, S. Zahra, J. Dumville

**Affiliations:** 1 York Trials Unit, Department of Health Sciences, Faculty of Science, University of York, Lower Ground Floor ARRC Building, York, YO10 5DD UK; 2 Centre for Health Economics, University of York, York, YO10 5DD UK; 3 Manchester University NHS Foundation Trust, Oxford Road, Manchester, M13 9WL UK; 4 Division of Nursing, Midwifery and Social Work, Jean McFarlane Building, University of Manchester, Oxford Road, Manchester, M13 9PL UK; 5 University of Hull, Hull York Medical School and Hull University Teaching Hospitals NHS Trust, Hull, UK; 6 Norfolk and Norwich University Hospitals NHS Foundation Trust, Colney Lane, Norwich, NR4 7UY UK; 7 NCS Woundcare Consulting Limited, Cornmill Lane, Leeds, LS17 9EQ UK

**Keywords:** Compression therapies, Time to healing, Venous leg ulcer, Randomised controlled trial, Wound healing

## Abstract

**Background:**

Venous leg ulcer(s) are common, recurring, open wounds on the lower leg, resulting from diseased or damaged leg veins impairing blood flow. Wound healing is the primary treatment aim for venous leg ulceration, alongside the management of pain, wound exudate and infection.

Full (high) compression therapy delivering 40 mmHg of pressure at the ankle is the recommended first-line treatment for venous leg ulcers. There are several different forms of compression therapy available including wraps, two-layer hosiery, and two-layer or four-layer bandages.

There is good evidence for the clinical and cost-effectiveness of four-layer bandage and two-layer hosiery but more limited evidence for other treatments (two-layer bandage and compression wraps). Robust evidence is required to compare clinical and cost-effectiveness of these and to investigate which is the best compression treatment for reducing time to healing of venous leg ulcers whilst offering value for money. VenUS 6 will therefore investigate the clinical and cost-effectiveness of evidence-based compression, two-layer bandage and compression wraps for time to healing of venous leg ulcers.

**Methods:**

VenUS 6 is a pragmatic, multi-centre, three-arm, parallel-group, randomised controlled trial. Adult patients with a venous leg ulcer will be randomised to receive (1) compression wraps, (2) two-layer bandage or (3) evidence-based compression (two-layer hosiery or four-layer bandage).

Participants will be followed up for between 4 and 12 months. The primary outcome will be time to healing (full epithelial cover in the absence of a scab) in days since randomisation. Secondary outcomes will include key clinical events (e.g. healing of the reference leg, ulcer recurrence, ulcer/skin deterioration, amputation, admission/discharge, surgery to close/remove incompetent superficial veins, infection or death), treatment changes, adherence and ease of use, ulcer related pain, health-related quality of life and resource use.

**Discussion:**

VenUS 6 will provide robust evidence on the clinical and cost-effectiveness of the different forms of compression therapies for venous leg ulceration.

VenUS 6 opened to recruitment in January 2021 and is currently recruiting across 30 participating centres.

**Trial registration:**

ISRCTN67321719. Prospectively registered on 14 September 2020

**Supplementary Information:**

The online version contains supplementary material available at 10.1186/s13063-023-07349-2.

## Administrative information

Note: the numbers in curly brackets in this protocol refer to SPIRIT checklist item numbers. The order of the items has been modified to group similar items (see http://www.equator-network.org/reporting-guidelines/spirit-2013-statement-defining-standard-protocol-items-for-clinical-trials/).

**Table:** 

Title {1}	A randomised controlled trial of compression therapies for the treatment of venous leg ulcers (VenUS 6): study protocol for a pragmatic, multicentre, parallel group, three arm randomised controlled trial
Trial registration {2a and 2b}.	ISRCTN 67321719 (https://doi.org/10.1186/ISRCTN67321719). Prospectively Registered: 14.09.2020 Recruitment Infographic SWAT - MRC Hub for Trials Methodology Research SWAT repository #116 Registered 13.04.2020 Retention Thank You Card SWAT - MRC Hub for Trials Methodology Research SWAT repository #119 Registered 13.04.2020 Retention Newsletter SWAT - MRC Hub for Trials Methodology Research SWAT repository #28 Registered 01.07.2007 Retention Pen SWAT - MRC Hub for Trials Methodology Research SWAT repository #92 Registered 01.04.2019
Protocol version {3}	V1.5 26.05.2022
Funding {4}	This project was funded by the National Institute for Health Research (NIHR) Health Technology Assessment Programme (Project Reference: 128625). The views expressed are those of the author(s) and not necessarily those of the NIHR or the Department of Health and Social Care.
Author details {5a}	^1^York Trials Unit, Department of Health Sciences, Faculty of Science, University of York, YO10 5DD. ^2^Division of Nursing, Midwifery and Social Work, Jean McFarlane Building, University of Manchester, Oxford Road, Manchester, M13 9PL. ^3^Centre for Health Economics, University of York, YO10 5DD. ^4^Manchester University NHS Foundation Trust, Oxford Road, Manchester M13 9WL ^5^University of Hull, Hull York Medical School and Hull University Teaching Hospitals NHS Trust, Hull, UK ^6^Norfolk and Norwich University Hospitals NHS Foundation Trust, Colney Lane, Norwich NR4 7UY ^7^NCS Woundcare Consulting Limited, Cornmill Lane, Leeds LS17 9EQ
Name and contact information for the trial sponsor {5b}	Manchester University NHS Foundation Trust 1st Floor, Nowgen Centre, 29 Grafton Street, Manchester M13 9WU research.sponsor@mft.nhs.uk
Role of sponsor {5c}	Study sponsor and funder have had no role in study design nor in the collection, management, analysis, or interpretation of data. They will have no role in the writing of associated publications and the decision to submit papers for publication.

## Introduction

### Background and rationale {6a}

Venous leg ulcer(s) are common, recurring, open wounds on the lower leg, resulting from diseased or damaged leg veins that impair blood flow. Treatment and care for these ulcers are often delivered by nurses in community clinics, out-patient settings, or peoples’ homes. Wound healing is the primary treatment aim for venous leg ulceration, alongside management of ulcer-related pain and wound exudate and infection prevention. When healing is achieved, the impaired blood flow can remain meaning those with healed ulcers are at high risk of recurrence.

Full (high) compression therapy delivering 40 mmHg of pressure at the ankle is the recommended first-line treatment for venous leg ulcers [1] although early access to endovenous ablation surgery has recently been shown to result in faster healing and is cost-effective [2, 3]. There are several different forms of compression therapy available to treat people with venous leg ulcers, including wraps, hosiery and bandages, but there is uncertainty about the relative clinical and cost-effectiveness of different options.

The Venous Leg Ulcer Study 6 (VenUS 6) is a three-arm randomised controlled trial (RCT) comparing the following approaches to delivering full compression therapy to people with venous leg ulcers:

- Arm 1: Two-layer hosiery or four-layer bandage systems (termed here as evidence-based compression
- Arm 2: Two-layer bandage systems
- Arm 3: Compression wraps (*adjustable hook-and-loop-fastened compression*)

Treatments in the evidence-based compression arm have been evaluated previously in large randomised controlled trials. A network meta-analysis [4] combining individual patient data and aggregate data from randomised controlled trials with 858 participants (including 387 VenUS 1 participants [5, 6]) showed that the four-layer bandage system reduced time to ulcer healing compared with the short stretch bandage. VenUS 1 reported the four-layer bandage system to be cost-effective. VenUS IV (457 participants) showed that two-layer hosiery is as effective as the four-layer bandage in healing venous leg ulcers, with two-layer hosiery the more cost-effective treatment (under a scenario assuming two-layer bandage and four-layer bandage had equal effectiveness) [7]. However, two-layer hosiery is not a suitable treatment for all patients, for example where the patient has oedematous legs.

Evidence is limited for the other two compression therapies being evaluated in VenUS 6: the two-layer bandage system and compression wraps. At the time of trial design, three randomised controlled trials (299 participants) [8–10] had compared two-layer bandage systems with four-layer bandage systems and there was uncertainty about the relative effects of the two treatments: risk ratio 1.17 (95% CI 0.87 to 1.57), resulting in a GRADE assessment of low certainty evidence. There is also limited randomised controlled trial evidence (24 participants) assessing compression wraps in this patient population. One study assessed the effectiveness of wraps for ulcer area reduction over a short period of follow-up (12 weeks) [11], and one assessed the costs of compression wraps [12]; however, time to healing was not reported in either study. Despite this limited evidence, two-layer bandages and compression wraps are increasingly reported as a treatment for venous leg ulcers.

### Objectives {7}

The key objectives of VenUS 6 are:

- To compare compression wraps with evidence-based compression for time to healing of venous leg ulcers
- To determine whether two-layer bandage systems are non-inferior to evidence-based compression for time to healing of venous leg ulcers
- To determine which full compression treatment for venous leg ulcers is cost-effective (i.e. provides the best value for money in routine use in the United Kingdom National Health Service (UK NHS)).

VenUS 6 will also compare the effect of compression wraps with evidence-based compression and two-layer bandage systems on ulcer recurrence, reported adverse events, ulcer-related pain, health related quality of life and adherence to treatment.

### Trial design {8}

VenUS 6 is a pragmatic, multi-centre, three-arm, parallel-group, randomised controlled, trial. Participants will be randomised in a 1:1:1 ratio to the three treatment arms.

Funded as part of the trial, three nested studies within a trial (SWATs) are included to assess the effectiveness of strategies to improve recruitment or retention:

1. SWAT 1 tests use of an infographic intervention to aid recruitment. This recruitment SWAT is cluster randomised (at the site level) with participants receiving either an infographic (visual document explaining the study) plus the standard participant information sheet (PIS) or just the PIS alone.
2. SWAT 2 tests use of a newsletter and/or thank you card (vs no newsletter and/or thank you card) to improve retention. The newsletter and/or thank you card are sent to participants at months four and nine following randomisation.
3. SWAT 3 tests the inclusion of a pen vs no pen with the month three questionnaire as a method of improving retention.

Participants will not be informed about the three SWATs included in this trial and therefore cannot provide their informed consent for their involvement. Due to the nature of the SWAT interventions, this approach is deemed low risk.

The trial also contains a process evaluation to better understand and explain the trial findings and potential mechanisms of impact. Trial data will be considered alongside data collected from semi-structured interviews exploring the views and experiences of participants and nurses throughout the trial. The protocol for this component will be reported separately (unpublished) and therefore is not detailed further here.

## Methods: participants, interventions and outcomes

### Study setting {9}

Participants will be recruited from up to 35 enrolled sites (acute NHS hospitals, community NHS trusts and primary care centres) in the UK. A list of enrolled sites is available in Supplementary File 1.

### Eligibility criteria {10}

VenUS 6 will include adult patients with a venous leg ulcer who meet all the inclusion criteria, and none of the exclusion criteria.

#### Inclusion criteria

1. Aged 18 years or over.
2. Has at least one venous leg ulcer, defined as ‘as any break in the skin on the leg which is either (a) is venous in appearance and accompanied by signs of chronic venous disease or (b) occurs in a person with a history of venous leg ulceration. The ulcer should be purely venous where clinically no other aetiology is suspected’.
  Note: The venous leg ulcer must lie wholly or partially within the gaiter region of the leg; venous leg ulcers which lie partially within the gaiter region and extend onto the foot will be included; however, venous leg ulcers that are confined to the foot only will not be eligible for inclusion.
3. Has an ankle–brachial pressure index (ABPI) of ≥ 0.8, taken within the previous 3 months or, where an ABPI measure is not possible, use of locally approved alternative assessments to rule out peripheral arterial disease, i.e. pulse palpation and Doppler auscultation, toe pressure assessment or arterial imaging, also taken within the last 3 months.
4. Is able to tolerate full compression.

#### Exclusion criteria

1. Is not willing to wear full compression
2. Has leg ulcers of non-venous aetiology or has significant peripheral vascular disease that contraindicates the use of full compression
3. Has ulcers confined to the foot
4. Lacks capacity or willingness to provide consent to participate in the trial
5. Is currently participating in another study evaluating treatments for their venous leg ulcer
6. Has a known allergy to any trial product
7. Has previously been recruited to VenUS 6
8. Is deemed to be clinically inappropriate to take part in the trial (at clinician’s discretion)
9. Has planned treatment to close/remove incompetent superficial veins (e.g. via endovenous ablation, sclerotherapy) within 28 days

### Informed consent {26a}

Informed consent will be obtained by a suitably qualified and experienced member of the research or clinical care team who has been authorised to do so by the Chief or Principal Investigator. The participant must personally sign and date the latest approved version of the informed consent form before any study-specific baseline procedures are performed.

Part of the consent process will also be to ask for consent to potentially follow up participants beyond the end of the trial for a maximum of 5 years. This is to allow future longer-term research to be considered. The consent form will also ask participants if they would like to take part in a semi-structured interview, which forms part of the study’s process evaluation. This is optional and not participating in the interviews will not affect main trial participation. Once informed consent has been obtained, baseline data will be collected.

### Additional consent provisions for collection and use of participant data and biological specimens {26b}

There are no biological specimens collected within VenUS 6; therefore, additional consent for collection and use is not required.

### Interventions

#### Explanation for the choice of comparators {6b}

Comparators were selected as compression wraps and two-layer bandage systems given the current lack of robust evidence for the clinical and cost-effectiveness of these treatments. Evidence-based compression was selected as the ‘control’ treatment arm as it is supported by the most robust evidence base [5–7].

#### Intervention and control descriptions {11a}

Regardless of treatment allocation, all participants will receive standard care including dressing changes, as per routine clinical practice and applied in accordance with manufacturer’s guidelines. The use of any primary contact dressing under the compression therapies being evaluated will be permitted.

##### Compression wraps arm

Any adjustable compression sleeve secured with hook and loop (Velcro™) fastenings, designed and marketed to be worn on the lower leg and foot, which aims to deliver > 40 mmHg of compression at the ankle with a system to guide this and is CE marked and available via NHS Prescription, will be permitted for use in VenUS 6.

The compression wraps may be used with a compressive or non-compressive liner and/or with the use of foot compression elements at the discretion of the treating health professional, provided the above criteria are met. Compression wraps marketed solely for treatment of lymphedema will not be included.

##### Evidence-based compression arm

Given the results of VenUS IV [7], which demonstrated two-layer hosiery is as effective as the four-layer bandage in healing venous leg ulcers, a choice comparator arm is included where allocated participants are offered two-layer compression hosiery, if they are deemed suitable, or a four-layer bandage system. This decision will be based on clinical judgement and participant preference. Any four-layer system delivering > 40 mmHg compression at the ankle will be permitted for use.

Similarly, any recognised two-layer compression hosiery delivering sustained graduated compression of > 40 mmHg at the ankle will be permitted for use. Two-layer hosiery comprises an initial layer of understocking or liner providing light compression over which a second overstocking (i.e. UK class 2 or 3 depending on understocking) is applied. Product-specific measurement tables will be consulted to ensure the participant receives the correct size kit, depending on foot length and ankle and calf circumference. Made to measure hosiery kits will also be permitted.

##### Two-layer bandage arm

Any recognised two-layer bandage system, consisting of an initial bandage layer covered with a top cohesive compression bandage, which aims to deliver > 40 mmHg compression at the ankle, will be permitted for use. This includes K-Two (Urgo), Coban 2, Andoflex and Actico2c. Other two-layer bandage kit systems will be considered on a case-by-case basis by the Chief Investigator, Trial Manager, and clinical members of the trial management team prior to use.

#### Criteria for discontinuing or modifying allocated interventions {11b}

Given the pragmatic nature of the trial, the decision for discontinuation of the intervention or control treatment will be made by the clinical care team in conjunction with the participant. Details of discontinuation and any alternative treatments provided will be recorded during ulcer-related dressing visits and/or monthly follow-up calls.

During the study, modifications may be made to the treatment as required by the clinical care team and details of any changes will be recorded during ulcer-related dressing visits and/or monthly follow-up calls.

#### Strategies to improve adherence to interventions {11c}

Decisions for continuation or discontinuation of interventions will be at the discretion of the clinical care team, in conjunction with the participant, so no specific strategies have been included to improve intervention adherence.

#### Relevant concomitant care permitted or prohibited during the trial {11d}

Throughout the study, concomitant medications or treatments deemed necessary may be prescribed.

#### Provisions for post-trial care {30}

At the end of the trial, participants will return to the care of their treating healthcare professional to determine any further treatment required. This may or may not include compression therapy as appropriate.

### Outcomes {12}

Healthcare professionals will phone participants monthly to monitor when the reference ulcer (defined as the ulcer with the largest surface area (cm^2^) where multiple ulcers are present) is healed. Other outcomes assessed in this way are the reference leg being ulcer-free, participant trial status and any clinical events experienced. Calls will continue throughout the study regardless of healing up until the participant exits the trial.

#### Primary outcome

The primary outcome for VenUS 6 will be time to healing of the reference ulcer, defined as ‘complete epithelial cover in the absence of a scab (eschar) with no dressing required’ in days from randomisation. Treating nurses will be asked to report the date when they consider the reference ulcer to be healed.

Once healing has been confirmed the treating nurse or participant will take a digital photograph once a week over the next four consecutive weeks. Standardised study specific, photography guidance and a camera will be provided to facilitate this.

Given the increased risk of bias associated with non-blinded outcome assessment for subjective outcomes such as ulcer healing [13], blinded outcome assessment will be undertaken using digital photographs. These photographs will be assessed independently by two clinical experts (experienced tissue viability nurses not involved in the care of VenUS 6 participants) blinded to allocation. Any disagreements will be resolved through a third reviewer. The blinded assessment of the healing date will be used as the primary healing endpoint. Non-blinded assessment of healing will be used as a secondary outcome.

#### Secondary outcomes

Secondary outcomes are:

1. Clinical events including healing of the reference leg, ulcer recurrence, ulcer/skin deterioration, amputation, admission/discharge, planned treatment to close/remove incompetent superficial veins (e.g. via endovenous ablation, sclerotherapy) within 28 days, infection, new ulcer occurrence or death. Details of any clinical events will be recorded during ulcer-related treatment visits and via the monthly telephone follow-up.
2. Changes to allocated treatment and reasons for change will be collected until either the participant’s reference leg is ulcer free or until the participant exits the trial. The date of visit, the type of compression being received and the type of primary contact dressing being used will be recorded. Where changes to the type of compression are made, we will record the date of the change, the changes made, the reason for this change and who requested the change (patient or clinician). Details will be recorded during ulcer-related treatment visits.
3. Health-related quality of life will be collected using the VEINES QoL [14] and EQ-5D-5L [15] which will be completed by the participant at baseline and 3, 6 and 12 months, with the EQ-5D-5L also collected at 1 month post-randomisation.
4. Adherence to treatment and ease of use questionnaires will be completed by the participant at 1, 3, 6 and 12 months, including views on the compression treatment received, volume of treatment use and reasons for reduced dose.
5. Ulcer-related pain using the 21-point Box Scale (BS-21) divided into units of five ranging from 0 (no pain) to 100 (the worst pain imaginable). This will be completed by the participant at baseline and 1, 3, 6 and 12 months.
6. Resource use, i.e. ulcer-related consultations received from the NHS, will be completed by participants at 3, 6 and 12 months. Details of ulcer-related dressing changes (frequency, type, activity) will also be collected at every nurse visit.

Additionally, data including demographics, diabetes status, venous leg ulcer surgical history, reference ulcer assessment, physical measures (i.e. stature, mass, and mobility), current ulcer treatments and an ulcer photograph will be collected at baseline.

#### SWAT outcomes

The primary outcome of SWAT 1 (use of infographic at recruitment) will be the recruitment rate, i.e. the proportion of participants in each group who are randomised into the host trial. Secondary outcomes will include the proportion of patients in each group who are screened but do not go on to be randomised, and the cost-effectiveness of the intervention.

For SWAT 2 (newsletter and/or thank you card), the primary outcome will be the questionnaire response rate, i.e. the proportion of participants who return their completed questionnaires at month six in each group. Secondary outcomes will include response rates at 12 months, whether a reminder notice is required, completeness of response, and cost of the intervention per participant retained.

For SWAT 3 (pen with questionnaire), the primary outcome will be the questionnaire response rate, i.e. the proportion of participants who return their completed questionnaires at month three in each group. Secondary outcomes will include response rates at 6 and 12 months, whether a reminder notice is required, completeness of response, and cost of the intervention per participant retained.

### Participant timeline {13}

**Fig. 1:**
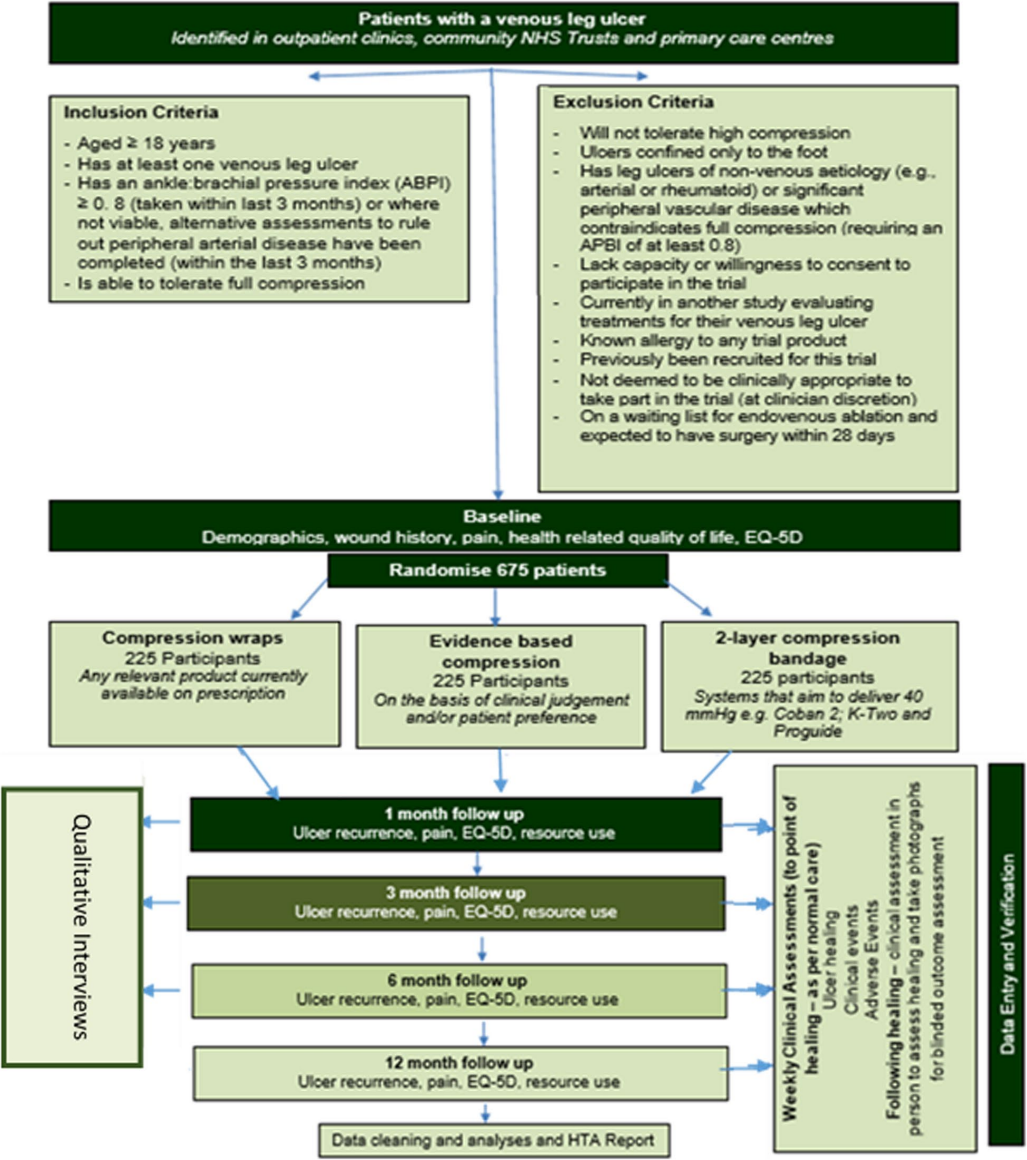
VenUS 6 participant timeline

See the participant timeline in Fig. 1 and SPIRIT figure in Table 1.

**Table 1:** VenUS 6 SPIRIT figure

	**Enrolment**	**Allocation**	**Clinical assessments**			**Postal questionnaires**				**GP check**
Timepoint	Pre-randomisation/baseline	Randomisation	Participant visit log (weekly)	Participant events form (monthly and when required)	Telephone calls (monthly)	1 mon	3 mon	6 mon	12 mon	
Enrolment										
Eligibility screen	✓									
Informed consent	✓									
Baseline data	✓									
Allocation		✓								
Staff collected										
Wound Healing			✓	✓	✓					
Changes to treatment			✓							
Other clinical events			✓^a^	✓	✓					
Adverse events			✓	✓	✓					
Confirmation of death				✓	✓					✓
Participant-reported										
HRQOL,	✓					✓	✓	✓	✓	
Resource use							✓	✓	✓	
Wound-related pain; participant adherence; ease of treatment use						✓	✓	✓	✓	

### Sample size {14}

Based on parameters from VenUS I [5, 6] and VenUS IV [7], a hazard ratio (HR) of 1.33 will be used as the non-inferiority margin for the comparison of the two-layer bandage arm and evidence-based compression arm. We assume a median time to healing of 2.3 months in the evidence-based compression group, an average follow-up time of 12 months and 10% attrition (pre-healing). We also assume that there is truly no difference between evidence-based compression and two-layer bandage under the alternative hypothesis for this test (i.e. HR = 1). Under these assumptions, 225 patients per group are required to obtain 80% power for a one-sided test of size 2.5% of the null hypothesis that two-layer bandage is inferior to evidence-based compression by a clinically relevant amount (i.e. HR = 1.33).

We also plan to recruit 225 patients to the compression wraps group. Under the same assumptions regarding healing rate in the evidence-based compression group, length of follow-up time and attrition as stated above, and assuming a hazard ratio of 1.33 (comparing compression wraps with evidence-based compression) under the alternative hypothesis, this sample size (i.e. 225 per group) obtains 80% power for a superiority comparison of evidence-based compression and compression wraps using a two-sided test of size 5%. If the two-layer bandage and evidence-based compression groups are combined and compared 2:1 against compression wraps, then under the same assumptions as the superiority comparison outlined above, this sample size (i.e. 450 vs 225) would obtain 90% power for a two-sided test of size 5%.

We will only combine the evidence-based compression and two-layer bandage groups for the superiority comparison with compression wraps if two-layer bandage is found to be non-inferior to evidence-based compression (i.e. the null hypothesis of the non-inferiority comparison is rejected). If the null hypothesis of the non-inferiority comparison is not rejected, then the evidence-based compression and two-layer bandage groups will not be combined, and compression wraps will be compared with each of these groups individually (i.e. compared 1:1:1). Under the same alternative hypotheses (i.e. HR(evidence-based compression/two-layer bandage) = 1 and HR(compression wraps/evidence-based compression) = 1.33) and assumptions (i.e. median healing time of 2.3 months in the evidence based compression group, average follow-up time of 12 months, 10% attrition) as above, the power to detect superiority of compression wraps over evidence-based compression or evidence-based compression and two-layer bandage combined is approximately 86%.

For the SWATs, as is usual with nested trials, a formal power calculation to determine sample size has not been conducted as the sample size is constrained by the number of patients approached about, or recruited into, the study, respectively [16].

### Recruitment {15}

Potential participants will be identified through clinical caseloads and screened for eligibility in the participating acute, community or primary care sites. It is however anticipated that most participants will be recruited from community NHS Trust settings.

Potential participants will be approached with further study details, including a PIS by a member of the clinical care or research team. The participant will be allowed as much time as required to consider the information and what giving informed consent involves and will be given the opportunity to ask any questions to relevant parties.

It will be made clear to individuals that they are free to withdraw from the study at any time for any reason without prejudice to future care, and without giving a reason for withdrawal. Should new information arise during the study which may affect an individual’s willingness to take part, this will be reviewed for addition to the PIS and a revised consent form will be completed, as necessary.

A recruitment SWAT is also included in the study, as previously described.

## Assignment of interventions: allocation

### Sequence generation {16a}

Independent 1:1:1 random allocation to the two treatment arms will be used. This will be via blocked randomisation (with randomly permuted blocks of varying sizes) and stratified by the following variables given these are known predictors of healing.

1. Ulcer duration (≤ 6 months and > 6 months)
2. Ulcer area (≤ 5cm^2^, > 5 cm^2^)

For SWAT 1, cluster randomisation (at the site level) will be used to reduce cross-contamination. Participants will be randomised 1:1 to the two arms of this SWAT.

For SWAT 2, participants will be allocated 1:1:1:1 using randomly varying block randomisation stratified by host trial treatment arm. For SWAT 3, similar randomisation will be used with an allocation ratio of 1:1.

### Concealment mechanism {16b}

Randomisation for the main trial will be completed by a centralised secure randomisation service hosted by York Trials Unit (YTU), University of York. Randomisation will be completed via the Internet, with information recorded to check eligibility prior to randomisation.

### Implementation {16c}

The allocation sequence for the main trial will be generated by the trial statistician who is independent to the recruiting teams at participating sites. This sequence will be implemented using the secure randomisation service that can be accessed by staff recruiting participants and will assign participants to one of the three trial arms.

Similarly for the nested recruitment and retention SWATs, generation of the allocation sequence will be undertaken independently by a researcher not involved with the recruitment of participants.

## Assignment of interventions: blinding

### Who will be blinded {17a}

As the study treatments cannot be adequately concealed, neither the trial participants nor the clinical care or research teams will be blinded to treatment allocation.

To limit potential bias in relation to primary outcome assessment, photographs taken of the wound following healing will be used to facilitate additional outcome (time to healing) verification by clinically experienced, independent, blinded observers.

For the three nested sub-studies, as participants will not be informed of these methodological studies, they will not be able to provide informed consent for their involvement and will therefore be blinded to their embedded trial allocation.

### Procedure for unblinding if needed {17b}

As study treatments cannot be blinded, there is no requirement for an unblinding procedure in this study.

Given the low risk of the nested sub-studies, there is no requirement for an unblinding procedure for these components either.

## Data collection and management

### Plans for assessment and collection of outcomes {18a}

Data will be collected at baseline and at 1, 3, 6 and 12 months post-randomisation, at routine ulcer dressing and compression clinical review appointments and by monthly telephone call. The maximum period for trial follow-up will be 12 months following randomisation; however, variable follow-up will be employed, and some participants may be followed up for less time (minimum four months).

At baseline, epidemiological data will be collected including participant details and demographics, weight, height and mobility (general and ankle). Ulcer history will be collected, and an assessment of the ulcer undertaken. Participants will complete outcome measures including the VEINES QOL [14], EQ-5D-5L [15], ulcer-related pain and resource use. A photograph of the ulcer will also be taken.

During the trial, participants will receive routine review of their compression treatment as per local clinical practice. We will collect data on each of these visits until the participant’s reference leg is ulcer free and no more visits are required, or until the participant exits the trial. Date of visit, type of compression received, and primary contact dressing will be recorded, with reasons for the change from allocated compression and the date on which this occurred.

Treating nurses will report when they consider the reference ulcer, and the reference leg, has healed. Additionally, data on the reference ulcer will be collected via digital images taken by the treating nurse or participant at the point of ulcer healing and then once a week for the following 4 weeks. Wherever possible the healing visits will be completed face to face, by the research nurse, with the participant at home, or in a clinical care setting if preferred. If face to face contact cannot be facilitated for a visit, NHS-approved video call technology may be used, with a screenshot taken of the ulcer, or alternatively where this is not possible the participant will be asked to take and return a photograph of their ulcer. Study-specific guidance will be provided to both the nurses and participants to allow standardisation of images.

These images will be assessed by two clinical experts blinded to allocation to confirm the date of healing, with disagreements resolved through discussion and the involvement of a third reviewer if required. The blinded assessment of healing date will be used as the primary healing endpoint. Non-blinded assessment of healing will be used as a secondary outcome.

Monthly telephone calls will also be completed with the participant to assess reference leg healing, ulcer recurrence and clinical events, e.g. ulcer infection, ulcer deterioration, surgical intervention and death.

Data will be collected via participant-completed questionnaires at 1, 3, 6 and 12 months after randomisation. Self-reported data will include wound-related pain, treatment adherence and ease of use, health-related quality of life (VEINES-QoL and EQ-5D-5L) [14, 15] and resource use. Where no response is provided, a reminder letter will be sent after 2 weeks to encourage completion. Where no response is provided after a further 2 weeks, participants may be contacted by telephone to collect data.

### Plans to promote participant retention and complete follow-up {18b}

With the study primary outcome being time to ulcer healing, if participants wish to withdraw their involvement from the trial, we will seek, where possible, consent to obtain data on healing status from the participant’s treating healthcare professional. This should ensure attrition of the primary outcome data is limited.

As well as the two retention SWATs already described, a range of strategies will be used to maximise the amount of participant-reported questionnaire data collected. If there is no response to the initial postal questionnaire mailing, after 2 weeks a reminder letter and questionnaire will be sent and this will be followed 2 weeks later, if no response, with a telephone call to the participant with the aim of collecting data. Participants will also receive an unconditional £10 with the 12-month questionnaire. This strategy has previously been reported as effective in improving participant retention in relation to questionnaire response rates [17].

### Data management {19}

Participant data, required by the protocol, will be recorded on case report forms (CRF). Separate CRFs will be used to collect clinical information and patient-reported information.

To ensure high-quality data, data collected within the CRFs will be processed at the YTU (University of York), using a licensed, automated, electronic system (Teleform), which allows data to be entered, checked and validated. Further details of the processing of the data will be documented in a study-specific data management plan.

Study documentation will be retained in accordance with Good Research Practice and UK Law for 5 years after study completion in the Trial Master and Investigator Site Files, after which time information will be securely destroyed.

### Confidentiality {27}

Study data will be managed in keeping with GDPR legislation, Good Clinical Practice (GCP) research standards and the Data Protection Act 2018.

To ensure confidentiality, all participants will be allocated a unique coded ID number, which will be used to identify them throughout the trial.

Personal data held electronically will be stored on the study-specific participant management system which will record identifiable information and participant activity to enable study coordination. The system will be housed on YTU, University of York servers, which are secure and are subject to rigorous testing. Sites will have access to the system, via individual password, to facilitate randomisation, and permissions for access will also be detailed within the study delegation log. The study team based at YTU will have access to the system, via individual password, to facilitate study conduct. Permissions for access will also be detailed within the study delegation log.

Paper forms containing participant identifiable information (e.g. patient details form and consent form) will be held in a separate location to the questionnaire data. Identifiable information will be stored securely in a locked filing cabinet in an access-restricted office.

Photographs collected to record ulcer status will be anonymised prior to electronic transfer by sites, via the NHS Approved Digital Encryption guidance, to the University of York, where they will be stored on an encrypted and password-protected drive.

To ensure that the study is conducted correctly, participants’ data may be reviewed by members of the research team or other authorised people, always maintaining confidentiality. Participants will consent to this review of their data at the start of the study. Identifying information will be removed before the data is analysed and the results presented and published.

### Plans for collection, laboratory evaluation and storage of biological specimens for genetic or molecular analysis in this trial/future use {33}

There will be no biological specimens collected within the VenUS 6 trial; therefore, no plans are required for the collection, evaluation or storage of such specimens.

## Statistical methods

### Statistical methods for primary and secondary outcomes {20a,}

#### Statistical analysis

##### Analysis of the primary outcome: time to healing

**Fig. 2:**
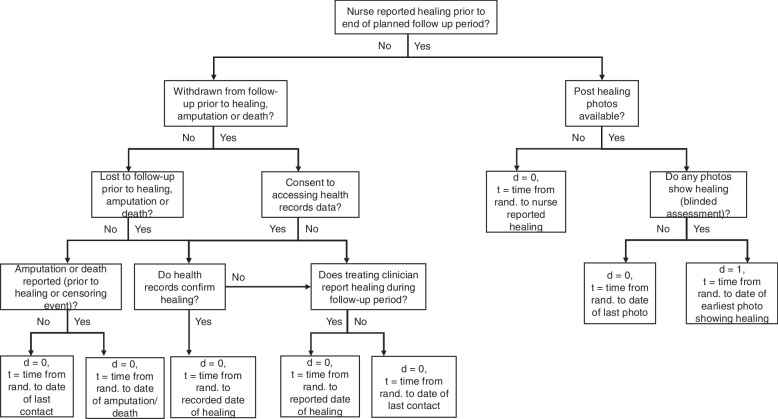
VenUS 6 censoring of healing

The trial analyses will follow a detailed pre-specified statistical analysis plan (SAP), which will be approved and made available on the funder’s website before the end of patient recruitment. The primary outcome will be time to healing of the reference ulcer as defined in the ‘Primary outcome’ section. If, prior to healing of the reference ulcer, participants reach the end of study follow-up, are lost to follow-up, withdraw from follow up and do not consent to continued healthcare records access for healing data or experience competing events (death or amputation of the reference leg), healing times will be censored as detailed in Fig. 2.

The primary analyses will include both non-inferiority and superiority comparisons. For the non-inferiority comparison (two-layer bandage vs evidence-based compression), two treatment effect estimands will be targeted.^1^ An unconstrained treatment policy estimand whereby all intercurrent events (e.g. treatment switches/cessations and receipt of surgery) will be accepted as part of completely pragmatic treatment policies (essentially an intention-to-treat analysis) and a constrained estimand whereby certain constraints are placed on the intercurrent events that are accepted as a part of the treatment policies.

Intercurrent events (such as treatment switches) will often result in the care received by patients allocated to different treatment groups being more similar, potentially resulting in attenuation of differences between treatment groups. Hence, estimands that do not address the influence of intercurrent events are potentially anti-conservative in the context of investigating non-inferiority. This provides the rationale for the constrained treatment policy estimand; the purpose of this estimand is to remove the influence of intercurrent events that could either be mitigated in practice (e.g. treatment switches that could be avoided by appropriate use of analgesia), or that might not be applicable to all patients (e.g. surgical treatments that some patients cannot/will not receive), whilst accepting the influence of intercurrent events that cannot be easily avoided as an unalterable part of the treatment strategies being compared (e.g. treatment switches due to allergy/adverse events). For the superiority comparisons, just the unconstrained treatment policy estimand will be targeted. Precise description of the unconstrained and constrained treatment policy estimands, and the estimators/methods that will be used to estimate them, will be given in the SAP.

For the non-inferiority comparison, the unconstrained treatment policy estimand will be estimated by modelling reference ulcer healing time using a Cox proportional hazards model with a three-level variable for group allocation (evidence-based compression vs two-layer bandage vs compression wraps), conditioning on the following baseline covariates: reference ulcer area, reference ulcer duration, participant age, participant mobility status and recruitment site (via shared frailties for participants recruited at the same site). For the constrained treatment policy estimand, patients will have their reference ulcer healing times censored at the first of any relevant departures from the constrained treatment policies. These healing times will then be analysed using a similar model as used to estimate the unconstrained treatment policy estimand. For both analyses, the point estimates and 95% confidence intervals (CIs) of the hazard ratios (HRs) for all between-group contrasts will be reported, together with estimated differences in median healing times (with 95% CIs based on non-parametric bootstraps). For each estimand, the estimated upper 95% confidence limits of the HRs for the evidence-based compression vs two-layer bandage contrasts will be compared with the non-inferiority margin of 1.33. If either of these upper limits is greater than 1.33, then the null hypothesis that two-layer bandage is inferior to evidence-based compression will not be rejected. If both estimates are less than 1.33, then the null hypothesis that two-layer bandage is inferior to evidence-based compression will be rejected.

If the null hypothesis that two-layer bandage is inferior to evidence-based compression is rejected, then a further model will be fitted with the two-layer bandage and evidence-based compression groups combined and compared with compression wraps (i.e. a two-level variable for group allocation—evidence-based compression + two-layer bandage vs compression wraps), with the same fixed effects and shared frailties as previously detailed. The point estimate and 95% Wald method CI of the HR for the two-layer bandage + evidence-based compression vs compression wraps contrast will be reported, together with the estimated difference in median healing time (with 95% CIs based on non-parametric bootstraps).

##### Analyses of secondary outcomes

Secondary time to event outcomes include time to healing of the reference leg and time to ulcer recurrence. Time to healing of the reference leg will be calculated and analysed in a similar manner to the primary outcome, although only the unconstrained treatment policy estimand will be targeted and there will be no combined two-layer bandage and evidence-based compression vs compression wraps comparison. Time to recurrence is defined as time from an ulcer-free reference leg to date of recurrence of a new ulcer on the reference leg. Participants whose reference leg heals and remains healed until they reach the end of follow-up or are withdrawn will have their time to recurrence censored at the relevant time, as will participants who experience a competing event (mortality and amputation of the reference leg). This outcome will be analysed using a similar model as used for the analyses of the primary outcome, although there will be no combined two-layer bandage and evidence-based compression vs compression wraps comparison.

Other secondary outcomes include health-related quality of life measures (VEINES and EQ-5D-5L [14, 15]), ulcer-related pain (at 3, 6 and 12 months) and adverse events. Descriptive statistics will be presented. Where appropriate continuous measures will be analysed using appropriate generalised linear regression models and categorical variables analysed using logistic regression or ordinal regression models. Summaries of the compression treatments received and adherence to the randomised treatment will be reported by allocation.

#### Economic analysis

We will explore the cost-effectiveness of all relevant compression systems in the treatment of venous leg ulcers by extending the VenUS IV cost-effectiveness modelling approach [7] to include up to date evidence including data from VenUS 6. This is likely to be more useful to decision-makers.

On this basis, and under a UK NHS and the Personal Social Services perspective [19], a decision analytic modelling approach will be taken. The decision model will aim at estimating the relevant costs and health benefits of all relevant compression systems, to fully inform judgements on the long-term cost-effectiveness of these interventions in the treatment of venous leg ulcers. The modelling exercise will aim to represent possible patient disease (or health) pathways and, by doing so, can incorporate multiple sources of evidence, extrapolating from limited data, and explore uncertainty over parameter values and structural assumptions. In this specific case, the main advantage of using a decision analytic model is in allowing the compression systems here to be compared to the wider set of relevant high compression treatments by including external information to the trial. The planned decision analytic model will extend previous work and include data from the network meta-analysis outlined above. Based on the selected model, further literature searching will be conducted to identify evidence on the following categories of model parameters: health-related quality of life/utility, costs and resource use, ulcer recurrence and mortality, to be used alongside current trial data.

Costs and health benefits conditional on healing status (health-related quality of life measured using EQ-5D-5L) will be derived using regression approaches, allowing for key covariates and uncertainty in estimates. Alternative scenarios regarding the extrapolation of the primary outcome over the lifetime of the model and the evidence informing it will be explored. Alternative scenarios exploring the extrapolation of secondary outcomes such as wound recurrence will also be explored. Uncertainty in the evidence base used to populate the decision analytic model will be characterised using appropriate distributions and any uncertainty in the adoption decision demonstrated using probabilistic sensitivity analysis. The value of further data collection using value of information analysis will be established.

#### SWAT analyses

##### SWAT 1

The primary analysis will be the difference in recruitment rates between those receiving the infographic in addition to the PIS and those not receiving the infographic. This will be analysed using a mixed effect logistic regression with a fixed effect for SWAT allocation and a random intercept for site. Logistic regression will also be used to assess the proportions per group of those responding to a recruitment invitation but who were not randomised.

The difference in cost per recruited participant between those offered the infographic and those not offered the infographic will be calculated. In addition to the direct costs of the infographic, the cost of staff time spent administering the recruitment packs may also be included.

##### SWAT 2

The difference in retention rates at 6 months will be analysed using a mixed effect logistic regression model including each intervention (thank you card and newsletter) and main trial treatment allocation as fixed effects and site as a random intercept. Adjusted odds ratios and corresponding 95% CIs will be obtained from this model. The presence of an interaction between the two interventions will also be tested using an interaction term.

The difference in the proportion of participants requiring a reminder letter mailing will be analysed using a similar model to the primary outcome. The difference in completeness of responses at 6 months will be analysed using a proportional odds model using similar adjustments to the primary outcome.

The difference in cost per retained participant between those sent a thank you card and/or newsletter and those not sent the thank you card and/or newsletter will be calculated. In addition to the direct costs of the thank you card, newsletter and postage, it may also be necessary to include the cost of staff time spent administering the mail out (for example filling and labelling envelopes).

The secondary outcomes at 12 months will be analysed as described above for the 6-month outcomes.

##### SWAT 3

The difference in retention rates at 3 months will be analysed using a mixed effect logistic regression model including each intervention (pen/no pen) and main trial treatment allocation as fixed effects and site as a random effect. Adjusted odds ratios and corresponding 95% CIs will be obtained from this model.

The difference in the proportion of participants requiring a reminder letter mailing will be analysed using a similar model to the primary outcome. The difference in completeness of responses at 3 months will be analysed using a proportional odds model using similar adjustments to the primary outcome.

The difference in cost per retained participant between those sent a pen and those not sent the pen will be calculated. In addition to the direct costs of the pen and postage, it may also be necessary to include the cost of staff time spent administering the mail out (for example filling and labelling envelopes).

The secondary outcomes at 12 months will be analysed as described above for the 6-month outcomes.

### Interim analyses {21b}

There are no planned interim analyses and no planned stopping rules for this trial.

### Methods for additional analyses {20b}

Exploratory analyses will be undertaken to assess whether reference ulcer size and duration are together a possible source of treatment effect heterogeneity. If two-layer bandage is found to be non-inferior to evidence-based compression, then the subgroup investigations will be conducted using both the three-treatment group model, and the two-treatment group model. If two-layer bandage is found to be inferior, then only the three-treatment group model will be used for subgroup investigations.

In either case, a similar model(s) to the primary analysis will be fitted, but with a term for Margolis Index score at baseline (see Table 2) and all two-way interactions between treatment group and Margolis Index score added to the linear predictor [11]. The point estimates of the hazard ratios for allocation within each Margolis Index subgroup will be presented together with Wald method 95% confidence intervals. The reference ulcer Margolis Index score for participant $i$ at recruitment site $j$ at baseline is defined as follows:

**Table 2:** Margolis Index score definition

**Margolis Index score**	**Definition**
0	Reference ulcer area ≤ 5cm^2^ and reference ulcer duration ≤ 6 months
2	Reference ulcer area > 5cm^2^ and reference ulcer duration > 6 months
1	Otherwise

### Methods in analysis to handle protocol non-adherence and any statistical methods to handle missing data {20c}

For the planned analyses of the time to event outcomes, we assume that censoring is non-informative, and therefore that the observed healing times adequately represent (conditional on the independent variable included) the healing times of those that have their healing times censored. We also assume that any missing baseline covariate data (e.g. log (baseline ulcer area)) is missing completely at random and impute any missing values using conditional mean imputation (details will be included in the SAP). For the analyses of ulcer-related pain and VEINES scores, we will use multiple imputation to address missing covariate or outcome data, if the proportion of randomised cases excluded from these analyses is greater than 10%.

### Plans to give access to the full protocol, participant-level data and statistical code {31c}

This document constitutes the full protocol. Datasets and statistical code used in this study will be available from the corresponding author on reasonable request following completion of the trial.

## Oversight and monitoring

### Composition of the coordinating centre and trial steering committee {5d}

The coordinating team will comprise the sponsor and Chief Investigator (based at the University of Manchester) and the trial manager, trial coordinators, data management and administrative support (based at YTU, University of York). The coordinating team will ensure all relevant approvals are in place, will train and support sites to undertake the study and will put measures in place to obtain accurate data. The data management team will process and check data against validation criteria agreed with the trial manager.

Independent study oversight will be provided by a Trial Steering Committee (TSC), which will monitor the progress of the study and provide independent advice at routine meetings. The TSC will comprise non-partisan clinicians, experienced health service researchers, trial statistician and study sponsor. An independent patient representative (who has received direct treatment for a venous leg ulcer within the NHS) will form part of the TSC. This member acts as a representative for the patient and public involvement (PPI) group on the TSC. Additionally, this member is the foreperson and collaborator of the Venus 6 patient advisory group (PAG), relaying qualitative patient feedback to the TSC on various study matters.

### Composition of the data monitoring committee, its role and reporting structure {21a}

The study will be regularly reviewed by the Data Monitoring Committee (DMC), comprising of independent clinicians and health service researchers with appropriate expertise. The DMC will be independent from the sponsor. The DMC will monitor the data arising from the study and recommend to the TSC whether there are any ethical or safety reasons why the trial should not continue.

### Adverse event reporting and harms {22}

For the purposes of the VenUS 6 trial, adverse events will be defined as any untoward medical occurrence, experienced by a clinical trial participant and which is temporally associated with study treatment (interventions or control) and is related to the ulcer or to the study intervention or control treatments. Depending on their seriousness (not severity), adverse events will be sub-categorised as serious adverse events (SAE) and non-serious adverse events (AE).

Adverse events, which might be expected, include skin maceration, ulcer deterioration, wound-related infection, bandage-/hosiery-related pain/discomfort, dryness, excoriation, non-SAE medical event, occurrence of new ulcer, skin damage, skin deterioration and ulcer-related pain.

Serious adverse events (SAE) will be defined as any untoward medical occurrence that:

1. Results in death
2. Is life-threatening
3. Requires unplanned inpatient hospitalisation or prolongation of existing inpatients’ hospitalisation
4. Results in persistent or significant disability or incapacity
5. Is a congenital anomaly or birth defect
6. Any other important medical condition that, although not included in the above, may require medical or surgical intervention to prevent one of the outcomes listed.

Where an adverse event is reported, participating sites will be required to promptly report this to YTU. Where the event is considered serious, causality and expectedness will be confirmed by the Chief Investigator or another clinical member of the trial management team. All events will be followed up until the event resolves or a decision is made for no further follow-up. Participants experiencing SAEs which are deemed to be related to the trial treatments (intervention or control) and which remain ongoing at the time of participant trial exit will be followed up for one further month beyond trial exit.

Where events are unexpected and related these will be reported to the research ethics committee and Sponsor within 15 days.

Adverse and serious adverse events will be routinely reported to the DMC and TSC for their review and oversight. Where repeated adverse events (serious or non-serious) of a similar type are observed, these will be discussed with the DMC and will be onward reported to the Research Ethics Committee (REC) and Sponsor should concerns be raised in relation to the type of event and/or frequency observed.

### Frequency and plans for auditing trial conduct {23}

No on-site auditing of trial conduct will be completed, unless circumstances prevail (e.g. serious breach of GCP) that warrant this. Centralised monitoring checks of eligibility and consent will be completed for 100% of participants and an annual audit of site files and documentation, via a self-complete checklist, will be completed with each participating NHS Trust. Further details relating to these activities will be documented in a study-specific monitoring plan.

The TMG will meet every 2 months to continuously evaluate the conduct of the study, in addition to routine review by the independent DMC and TSC.

### Plans for communicating important protocol amendments to relevant parties (e.g. trial participants, ethical committees) {25}

Any protocol amendments will be approved by the Sponsor (Manchester University NHS Foundation Trust) and the Funder (National Institute for Health Research (NIHR) Health Technology Assessment Programme) prior to submission to the approving Research Ethics Committee (West of Scotland REC 4) and the Health Research Authority. Documentation will be provided to study sites for their local review and implementation as required.

### Dissemination plans {31a}

Following completion of the VenUS 6 Trial, irrespective of the magnitude of effect, we will submit the study results to peer-reviewed journals. A publication policy will be developed in advance detailing authorship, acknowledgments and review procedures for all publications resulting from VenUS 6 trial.

The executive summary and copy of the trial report will be sent to the National Institute for Health and Care Excellence (NICE). Integrated Care Boards and wider bodies will also receive copies, so that study findings can be translated into clinical practice nationally.

We will produce a Plain English Summary of the report and disseminate this to participants, members of our patient advisory group and relevant patient-focused websites. Patient information will also be generated for ‘Shared Decision Making’, the entry on Wikipedia and the Map of Medicine entry.

## Discussion

Use of compression wraps and two-layer bandage systems as treatments for venous leg ulcers have been increasing despite limited high-quality evidence to support their clinical and cost-effectiveness. Given the increasing use of these treatments, a randomised controlled trial comparing these treatments with the current gold standard compression treatments (evidence-based compression) is required; VenUS 6 is designed to generate the robust evidence required to fill this gap.

## Study status

Recruitment to VenUS 6 commenced in January 2021, and to date, 407 participants of the target 675 (60.3%) have been recruited. Thirty-one sites are currently open to study recruitment.

Results of VenUS 6 are currently expected in early 2024.

## Trial status

VenUS 6 Protocol v1.5 26.05.2022

Recruitment commenced on: 03.02.2021

Recruitment is currently expected to complete on 30.04.2023

## Supplementary Information

**Additional file 1.** VenUS 6 study sites.

**Additional file 2.** Participant consent form and Information sheet for completeness.

## Data Availability

This document constitutes the full protocol. Datasets and statistical code used in this study will be available from the corresponding author on reasonable request following completion of the trial.

## Abbreviations

ABPI: Ankle Brachial Pressure Index
AE: Adverse event
BMI: Body mass index
CACE: Complier Average Causal Effect
CI: Confidence interval
CRF: Case report form
DMC: Data Monitoring Committee
EQ5D5L: EuroQol 5 Domain 5 Level
GCP: Good Clinical Practice
HR: Hazard ratio
ID: Identification
NHS: National Health Service
NICE: National Institute for Health and Care Excellence
NIHR: National Institute for Health and Care Research
PAG: Patient Advisory Group
PIS: Participant Information Sheet
PPI: Patient and Public Involvement
QALY: Quality-adjusted life year
RCT: Randomised controlled trial
SAE: Serious adverse event
SWAT: Study Within A Trial
TMG: Trial Management Group
TSC: Trial Steering Committee
UK: United Kingdom
VLU: Venous leg ulcer
YTU: York Trials Unit

## Footnotes

1 An estimand is a precise description of the treatment effect to be estimated with regard to five key attributes: (1) population of interest, (2) treatment strategies being compared, (3) outcome of interest, (4) treatment effect summary measure and (5) handling of intercurrent events [18].
