# Supplementary material for: A randomised controlled trial of compression therapies for the treatment of venous leg ulcers (VenUS 6): study protocol for a pragmatic, multicentre, parallel-group, three-arm randomised controlled trial
Source: Trials. 2023 May 26;24:357. doi: 10.1186/s13063-023-07349-2 (PMC10223923; doi:10.1186/s13063-023-07349-2)
Supplement: Supplementary file 1 — Additional file 1. VenUS 6 study sites. [file 13063_2023_7349_MOESM1_ESM.docx]

| **Sites Open to Recruitment** | |
| --- | --- |
| Accelerate CIC | The Adam Practice |
| Birmingham Community Healthcare NHS Foundation Trust | Bradford Teaching Hospital NHS Foundation Trust |
| Cornwall Partnership NHS Foundation Trust | Derbyshire Community Health Services NHS Foundation Trust |
| Dorset Healthcare University NHS Foundation Trust | The Dudley Group NHS Foundation Trust |
| East Coast Community Healthcare CIC | Herefordshire and Worcestershire Health and Care NHS Trust |
| Hertfordshire Community NHS Trust | Hull University Teaching Hospital NHS Trust |
| Hywel Dda University Health Board | Kent Community Health NHS Foundation Trust |
| Lancashire and South Cumbria NHS Foundation Trust | Livewell Southwest (University Hospital Plymouth) |
| Manchester University NHS Foundation Trust | Midlands Partnership NHS Foundation Trust |
| Mid Yorkshire Hospitals NHS Trust | North Cumbria Integrated Care NHS Foundation Trust |
| Norfolk and Norwich University Hospitals NHS Foundation Trust | Northumbria Healthcare NHS Foundation Trust |
| The Shrewsbury and Telford Hospital NHS Trust | Solent NHS Trust |
| Somerset NHS Foundation Trust | Southern Health NHS Foundation Trust |
| University Hospital Birmingham NHS Foundation Trust | Walsall Healthcare NHS Trust |
| Wirral University Teaching Hospital NHS Trust | Your Healthcare CIC |
| **Sites Previously Recruited But Withdrawn** | |
| Cambridgeshire Community Services | Medway Community Healthcare |
| **Sites In Set Up** | |
| Countess of Chester Hospital NHS Foundation Trust | Newcastle Upon Tyne Hospitals NHS Foundation Trust |
| Sheffield Teaching Hospitals NHS Foundation Trust |  |

**Supplementary File 1: VenUS 6 Study Sites**
