# Supplementary material for: A randomised controlled trial of compression therapies for the treatment of venous leg ulcers (VenUS 6): study protocol for a pragmatic, multicentre, parallel-group, three-arm randomised controlled trial
Source: Trials. 2023 May 26;24:357. doi: 10.1186/s13063-023-07349-2 (PMC10223923; doi:10.1186/s13063-023-07349-2)
Supplement: Supplementary file 2 — Additional file 2. Participant consent form and Information sheet for completeness. [file 13063_2023_7349_MOESM2_ESM.zip › VenUS 6 Informed Consent Form V1.1 10.05.2021R1.pdf]

THE UNIVERSITY of York   VenUS 6 Venous Leg Ulcer Study   INSERT SITE LOGO

PARTICIPANT CONSENT FORM

Venous Leg Ulcer Study 6 (VenUS 6)

Participant ID: ☐☐☐☐

If you wish to take part in VenUS 6, **please place your initials in each of the boxes below, sign and date this form.**

*Please **initial** each box*

1. I confirm that I have read and understood the participation information sheet version …… dated …………………… for VenUS 6. I confirm I have had the opportunity to consider and discuss the information, to ask questions and get satisfactory answers where applicable. *Initials*

2. I understand that my participation is voluntary and I am free to withdraw from the study at anytime, without having to give any reason and without my medical care or legal rights being affected. *Initials*

3. I agree to the University of York holding copies of my consent form, other study related documents and my contact details to allow them to send me questionnaires. I understand that any information that could identify me will be kept strictly confidential and that no personal information will be included in the study report or other publication. *Initials*

4. I agree to my General Practitioner (GP) being informed of my participation in the study and being advised of any significant information in relation to my health that comes to light during my participation. *Initials*

5. I understand that relevant sections of my hospital/ GP medical notes and data collected during the study, may be looked at by individuals from the University of York Trials Unit, regulatory authorities, the Sponsor or the NHS Trust, where it is relevant to my taking part in this research. I give permission for these individuals to have access to my records. *Initials*

6. I consent to the storage, including electronic, of personal information collected for the study by [INSERT SITE NAME] NHS Hospital/ Trust, the Sponsor and University of York Trials Unit. I understand that any information that could identify me will be kept strictly confidential and that no personal information will be included in the study report or other publication. *Initials*

7. I understand that after this study has finished, data collected from me will be securely stored for a minimum of 5 years in accordance with requirements of the law after which arrangements for confidential destruction will take place. Information that could identify me will be kept strictly confidential and no personal information will be included in the study report or other publication. *Initials*

FUNDED BY NIHR | National Institute for Health Research

This study is funded by the National Institute for Health Research (NIHR) Health Technology Assessment Programme (NIHR128625). The views expressed are those of the author(s) and not necessarily those of the NIHR or the Department of Health and Social Care.

8. I understand that the information collected about me may be used to support other research in the future, and may be shared anonymously with other researchers. | Initials

9. I agree to have photographs taken of my leg ulcer(s) or the area of my leg where these ulcers were previously | Initials

10. **I agree to take part in VenUS 6** | Initials

OPTIONAL: If you also wish to take part in a VenUS 6 interview about your experiences of treatment, **please place your initials in each of the boxes below, sign and date this form**

11. I agree to take part in an interview exploring the experience I have had wearing compression therapies | Initials

12. I give consent to this interview being recorded, which may be used for education purposes, support future research and may be shared anonymously with other researchers. Personal information will never be associated with the recording. | Initials

13. I agree to the University of Manchester holding copies of my consent form, other study related documents and my contact details to allow them to conduct an interview. I understand that any information that could identify me will be kept strictly confidential and that no personal information will be included in the study report or publication. | Initials

14. I consent to the storage, including electronic, of personal information collected for the study by the Sponsor, University of Manchester and University of York Trials Unit. I understand that any information that could identify me will be kept strictly confidential and that no personal information will be included in the study report or other publication. | Initials

Print name	d d / m m / y y y y<br>__ __ / __ __ / 2 0 __ __	Signature
Name of participant (*please print*)	Date	Signature of participant
Print name	d d / m m / y y y y<br>__ __ / __ __ / 2 0 __ __	Signature
Name of person taking consent (*please print*)	Date	Signature of person taking consent

When completed: 1 for participant; 1 for researcher site file; 1 to be kept in medical notes
