# Supplementary material for: A randomised controlled trial of compression therapies for the treatment of venous leg ulcers (VenUS 6): study protocol for a pragmatic, multicentre, parallel-group, three-arm randomised controlled trial
Source: Trials. 2023 May 26;24:357. doi: 10.1186/s13063-023-07349-2 (PMC10223923; doi:10.1186/s13063-023-07349-2)
Supplement: Supplementary file 2 — Additional file 2. Participant consent form and Information sheet for completeness. [file 13063_2023_7349_MOESM2_ESM.zip › VenUS 6 Participant Information Sheet V1.4 05.09.2022R1.pdf]

THE UNIVERSITY of York

VenUS 6
Venous Leg Ulcer Study

[INSERT SITE LOGO]

Venous Leg Ulcer Study (VenUS 6)

A randomised controlled trial of compression therapies for the treatment of venous leg ulcers

You have been invited to take part in a research study.

Before you decide whether or not to participate it is important for you to understand why the research is being carried out and what it will involve.

Please take time to read the information carefully, discuss it with others and ask any questions you may have.

Summary of this study

- People with venous leg ulcers will routinely receive compression therapies as part of their standard care
- We are running this study to investigate the clinical and cost effectiveness for treating venous leg ulcers
- This study is comparing different compression therapies to discover which is the best treatment
- People participating will be asked to complete questionnaires for a minimum of 4 months and a maximum of 12 months (at 1, 3, 6 and 12 months) to be returned in freepost envelopes. We/you will also take photographs of the leg at certain points throughout the study to monitor progress

The study is being run across the United Kingdom and <<INSERT SITE NAME>> are running this study in your area.

The study is being run by the Universities of Manchester and York and Manchester University NHS Foundation Trust.

Contents

1. **Why are we doing this study?**
   - Why have I been invited to take part?
   - Do I have to take part?
2. **What would taking part involve?**
   - What happens if I no longer wish to take part?
3. **What are the possible benefits of taking part?**
4. **What are the possible disadvantages and risks of taking part?**
5. **More information about taking part**
   - What happens if something goes wrong?
   - What if new information becomes available?
   - Will taking part cost me money, and will I be paid?
   - Will taking part in this study be kept confidential
   - What happens when the research stops?
   - What will happen to the results of the study?
   - Who is organising and funding the research?
   - Who has reviewed the study?
   - What do I do now?
6. **How to contact us**

FUNDED BY
NIHR | National Institute for Health Research

This project is funded by the National Institute for Health Research Health Technology Assessment Programme (NIHR: 128625). The views expressed are those of the authors and not necessarily those of the NHS, the NIHR or the Department of Health and Social Care.

THE UNIVERSITY of York

VenUS 6
Venous Leg Ulcer Study

[INSERT SITE LOGO]

1. Why are we doing this study?

Venous leg ulcers are common, recurring open wounds on the lower leg.

Compression is the first line of treatment for venous leg ulcers. A range of compression therapies such as four-layer bandages, two- layer hosiery, two-layer bandages and compression wraps are regularly used in the NHS.

We are conducting this study because we are unsure whether one compression therapy is more effective at treating venous leg ulcers than the others. To find the answer to this question, VenUS 6 will recruit and gather important information from a large number of people with a venous leg ulcer(s).

Why have I been invited to take part in VenUS 6?

You have been invited to participate in this research study because you currently have a venous leg ulcer which requires treatment.

Do I have to take part?

No, taking part is completely voluntary. It is entirely up to you whether or not to be involved, and you are free to change your mind at any time. If you do decide to take part you will be given this information sheet to keep and you will be asked to sign a consent form to confirm your decision.

2. What would taking part involve?

Provided there are no health circumstances which would make it inappropriate for you to participate and you are happy to take part, we will ask you to sign a study consent form. You will be given a copy of this consent form, and copies will also be filed in your medical records and sent to York Trials Unit.

Once you sign the consent form, we will take a photo of one of your leg ulcer(s) and ask you some questions about your general health. You will then be **randomly allocated to one of three groups**. This means you will have an equal chance of receiving one of the compression treatments under investigation, in the same way that tossing a coin gives an equal chance of getting 'heads' or 'tails'. Your nurse and/ or doctor will not know which group you will be in until after you agree to take part. There is no reason to feel disadvantaged or disappointed by which treatment you are allocated to. All three treatments are regularly used in the NHS however we are running this study because at present there is not enough information to show that either treatment is any better than the other.

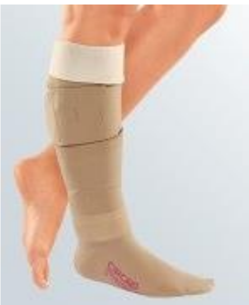

Compression wraps

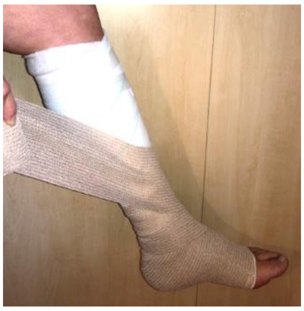

Four- layer bandage

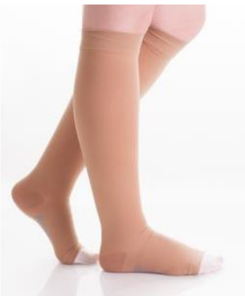

Two-layer hosiery

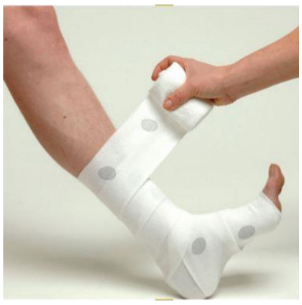

Two-layer bandage

Treatment

1. **Compression wraps** (adjustable hook-and-loop-fastened compression): A compression sleeve around the foot and leg
2. **Four-layer bandage or two-layer compression hosiery**: If you are allocated to this treatment group, you and your healthcare professional will decide which treatment you receive. This will be either four layers of compression bandages or two layers of compression stockings
3. **Two-layer compression bandage**: this bandage will comprise of an initial bandage layer with a top compression bandage

Additional information about these treatments will be provided to you by your nurse or doctor.

Routine Ulcer Treatment

You will continue to see your healthcare professional for your ulcer treatments to be carried out as normal. Your healthcare professional will check how the ulcer is healing and will record details of any changes to your treatment.

Once your ulcer has healed, this area of your leg will be photographed once a week for 4 weeks. Following the healing of your leg ulcer a nurse will also contact you once a month, by telephone, to check that your ulcer has not returned.

Study Questionnaires

Whichever treatment you receive, you will be asked to complete a questionnaire booklet at set time points (1, 3, 6 and 12 months), asking about your health and ulcer. We will send you these questionnaires in the post with a prepaid envelope for their return. As a small thank you for your time, we will send you a £10 voucher with the final questionnaire booklet which we ask you to complete.

Interview

As part of VenUS 6, we would also like to explore patient's experiences of using different compression therapies for venous leg ulcers. This is separate to the main study.

We will ask if you would be willing to take part in an interview to ask you some questions about compression treatments. If you are willing to take part, we may contact you to arrange an interview between 1 and 9 months after you start your compression treatment. The interview will last around one hour and we will record and transcribe the interview. Anonymised quotes may be used for publications or presentations. Please note we will not be able to include everyone who agrees to take part in the interview, so you may not hear anything regarding this participation.

If you agree to take part in an interview your consent form and contact details will be sent to the University of Manchester, in addition to York Trials Unit, for storage. You do not have to take part in this interview and if you choose not to it will not affect your participation in the main study or the ongoing care you will receive from the NHS.

Future Research

The information collected may be used to support other research in the future. Any information shared will be done so anonymously.

If I decide to participate, will my GP be notified?

With your consent, your GP will be notified that you are taking part in this study and for your safety, a record will be kept regarding your involvement.

What will happen if I do not want to carry on with the study?

If you agree to take part, but later change your mind, you can withdraw from the study at any time without giving reason. The care you receive currently or in the future will not be affected in anyway.

If you leave the study, we would like to use all the data we have collected from you, as this is important to the study. To safeguard your rights, we will use the minimum personally identifiable information possible.

3. What are the possible benefits of taking part?

Every effort will be made to improve your venous leg ulcer; however, we cannot say whether your ulcer will improve more quickly because of your participation in the study. The information we get from this study may have a significant impact upon helping people and healthcare professionals make more informed treatment choices in the future.

4. What are the possible disadvantages and risks of taking part?

Side effects for compression wraps, the two-layer bandage, four-layer bandages and the two-layer hosiery are uncommon and are treatments routinely used in the NHS for patients with venous leg ulcers. There are no anticipated increased risks through participation in this study. Being in the study will not harm or disadvantage your care in any way. Throughout your participation, you will continue to see your health care professional for your wound treatments to be carried out as normal.

5. More information about taking part

What happens if something goes wrong?

If you are concerned with any aspect of the study, you should speak to your treating healthcare professional or one of the researchers who will do their best to answer your questions (contact details below).

If you remain unhappy and wish to complain formally, you can obtain advice from your local Patient Advice and Liaison Service. Contact details are provided at the end of this leaflet.

In the unlikely event that something should go wrong and as a participant you wish to seek compensation, normal NHS negligence and insurance procedures apply. There is no special compensation system for the study. If you are harmed due to someone's negligence, then you should seek independent advice about any legal action. If you wish to complain, or have any concerns about any aspect of the way you have been approached or treated during the course of this study, the normal NHS complaints services are available to you.

In an emergency, you should contact your research doctor or nurse. The name of a contact nurse and telephone number where they can be reached is provided at the end of this leaflet.

What if new information becomes available?

Sometimes during a trial new information regarding the treatments being studied becomes available. If this happens, your healthcare professional will discuss with you and ask whether you want to continue in the study and if it is appropriate for you to continue to do so. If you decide to withdraw, your care will continue in line with routine practice. If you decide to continue, you will be asked to sign an updated consent form.

Will my taking part in the study cost me anything, and will I be paid?

Participation in this study should not cost you anything. We will collect information form you at your usual ulcer treatment visits and by sending questionnaires through the post, which will be returned in the freepost envelope provided. To thank you for your involvement in this study, we will send you £10 with the final questionnaire.

How will we use information about you?

We will need to use information from you and your medical records. This information will include your initials, NHS number, name, contact details and address. People will use this information to do the research or to check your records to make sure that the research is being done properly. People who do not need to know who you are will not be able to see your name or contact details. Your data will have a code number instead. Digital photographs of your ulcer, taken during the study will be transferred to York Trials Unit and anonymised as possible. If however, you have any identifying marks located close to your ulcer (e.g. a tattoo, a mole or scar) these may remain visible in the image.

The University of York will hold copies of your consent form, contact details (name, date of birth, address, telephone number and email address) and other study related documents to allow them to send you questionnaires. If you agree to take part in an optional interview these details will also be held by the University of Manchester (optional). All the information held about you will be kept in a safe and secure location.

Once we have finished the study, we will keep some of the data so we can check the results. We will write our reports in a way that no-one can work out that you took part in the study.

What are your choices about how your information is used?

You can stop being part of the study at any time, without giving reason, but we will keep information about you that we already have.

We need to manage your records in specific ways for the research to be reliable. This means we won't be able to let you see or change the data we hold about you.

Where can you find out more about how your information is used?

You can find out more about how we use your information

- At www.hra.nhs.uk/information-about-patients/
- By asking one of the research team

What happens when the research stops?

At the end of the study you will return to standard NHS care with your treatment nurse or doctor. If your ulcer does not fully heal during the study your care will continue with the treatment your doctor or nurse feels is best.

What will happen to the results of the study?

The results of the study will be made available to you once the trial has finished, and will be published in medical and nursing journals. Any interview quotes used will be fully anonymised. You will not be identified in any publication or presentation arising from this study.

Who is organising and funding the research?

This study is funded by the National Institute for Health Research Health Technology Assessment Programme (Project Number: NIHR128625). Your nurse and doctor are not personally receiving any money for conducting this study.

Manchester University NHS Foundation Trust (the Sponsor) is responsible for the conduct of this study, supported by York Trials Unit at the University of York and Professor Jo Dumville at The University of Manchester.

We will be using information from you and/or your medical records in order to undertake this research and the sponsor will act as the data controller for this study. This means that we are responsible for looking after your information and using it properly. Other collaborating institutions will also be processing your data and will keep identifiable information about you. You can find out more about how we use your information at: https://research.cmft.nhs.uk/getting-involved/gdpr-and-research

If you agree to take part in the trial, the University of York, and participating hospitals will keep data collected for a minimum of 5 years. Confidential destruction will then be arranged. Identifiable information collected about you (such as name, address, date of birth and contact details) will be confidentially destroyed at the end of the study.

Who has reviewed the study?

All research in the NHS is looked at by an independent group of people called a Research Ethics Committee. The aim of a Research Ethics Committee is to protect your interests. West of Scotland Research Ethics Service (Reference: 20/WS/0121) have reviewed and approved this study.

How have patients and public helped to design the study?

People affected by leg ulceration have confirmed the importance of this research question and have reviewed the participant information sheet and other study documentation

What do I do now?

After reading the information you may decide:

- ***Yes, I would like to take part:***
  If you are interested in taking part please discuss this study with your healthcare professional. You will be given a copy of this information sheet and asked to sign a consent form.
- ***No, I do wish to take part:***
  If you do not wish to take part you do not need to do anything, it will not affect the ongoing standard care you receive in any way from the NHS. Thank you for taking the time to read this information sheet.
- ***I am unsure and would like more information:***
  If you would like some further information please contact the nurse on the telephone number provided.

6. How to contact us

You are encouraged to ask questions if you wish before, during and after your treatment. If you have any questions, please contact us:

Principal Investigator: [INSERT SITE PI CONTACT DETAILS]

Research Nurse(s): [INSERT RESEARCH NURSE CONTACT DETAILS]

If you would like independent advice about whether or not to take part, or would wish to complain formally, the Patient Advice and Liaison Service (PALS) can be contacted on 0191 217 2996, or at ngccg.enquiries@nhs.net.

If you would like specific information about this study, you may contact VenUS 6 Trial Coordinators, venus6-trial-group@york.ac.uk Alternatively, you can contact Catherine Arundel, the Trial Manager on 01904 321 116 or at catherine.arundel@york.ac.uk. The chief investigator and overall lead for this study is Professor Jo Dumville who can be contacted at jo.dumville@manchester.ac.uk

Thank you for reading this information sheet and for considering taking part in this study
